# “That’s probably what my mama’s lungs look like”: how adolescent children react to pictorial warnings on their parents’ cigarette packs

**DOI:** 10.1186/s12889-018-6011-7

**Published:** 2018-09-15

**Authors:** Kaitlyn E. Brodar, M. Justin Byron, Kathryn Peebles, Marissa G. Hall, Jessica K. Pepper, Noel T. Brewer

**Affiliations:** 1 Department of Psychology, University of Miami, 5665 Ponce de Leon Drive, Coral Gables, FL 33146-0751 USA; 2 Department of Health Behavior, Gillings School of Global Public Health, University of North Carolina, 325 Rosenau Hall CB7440, Chapel Hill, NC 27599 USA; 3 Department of Family Medicine, School of Medicine, University of North Carolina, 590 Manning Dr., Chapel Hill, NC 27599 USA; 4 Lineberger Comprehensive Cancer Center, University of North Carolina, Chapel Hill, NC 27599 USA; 5 RTI International, 3040 E. Cornwallis Road, Research Triangle Park, NC 27709 USA; 6 Department of Epidemiology, School of Public Health, University of Washington, Seattle, WA 98195 USA

**Keywords:** Tobacco, Cigarettes, Pictorial warnings, Adolescents, Qualitative research

## Abstract

**Background:**

Pictorial cigarette pack warnings discourage smoking, but most evidence comes from studies of adults. Our qualitative study explored adolescents’ reactions to pictorial warnings on their parents’ cigarette packs.

**Methods:**

We interviewed 24 adolescents whose parents received pictorial warnings on their cigarette packs as part of a randomized clinical trial. We conducted a thematic content analysis of the interview transcripts.

**Results:**

Pictorial cigarette pack warnings led adolescents to imagine the depicted health effects happening to their parents, which elicited negative emotions. The warnings inspired adolescents to initiate conversations with their parents and others about quitting smoking. Adolescents believed the warnings would help smokers quit and prevent youth from starting smoking. Some current smokers said the warnings made them consider quitting.

**Conclusions:**

Conversations about the pictorial warnings may amplify their effectiveness for smokers, their adolescent children, and friends of the adolescent children. Cigarette pack warnings may reach a broad audience that includes adolescent children of smokers.

**Electronic supplementary material:**

The online version of this article (10.1186/s12889-018-6011-7) contains supplementary material, which is available to authorized users.

## Background

Adolescents, particularly those with parents who smoke, are a key population to target with tobacco prevention messages and policy interventions. Adolescents with a smoking parent are more likely to experiment with smoking at an early age and more likely to become regular smokers than those without a smoking parent [1]. There is a dose-response relationship, such that the adolescents’ likelihood of smoking increases with each additional year exposed to parental smoking. On the positive side, research indicates that parental smoking cessation may lower the risk of adolescent smoking initiation [2, 3].

Pictorial (graphic) warnings on cigarette packs are a promising approach for changing antecedents to smoking behavior and reducing smoking [4–7]. Most research on pictorial warnings comes from studies of adults. The existing studies of adolescents have largely been conducted in countries with active pictorial warning regulations [8–12]. One study of US adolescents indicated that high-emotion pictorial warnings increase perceptions of risk and intentions to quit compared to text-only warnings [13]. Another found that strong negative emotions mediate the relationship between perceived graphicness of cigarette warning labels and increased negative beliefs about smoking [14]. One limitation of past research on adolescents’ responses to pictorial warnings is that studies have been conducted almost exclusively in laboratory settings [15]. Also, there is a lack of research that specifically investigates how the vulnerable population of children of smokers react to pictorial warning labels. Thus, a gap remains in understanding how US adolescents, especially those whose parents are smokers, react to and think about pictorial warnings when exposed to them in a natural setting.

Our randomized clinical trial (RCT) of pictorial cigarette pack warnings among 2149 adult US smokers found that pictorial warnings led to more quit attempts and quitting than text-only warnings [5]. The RCT presented a unique opportunity to interview adolescent children of parents who participated in the trial. A quantitative study of these adolescents found that exposure to pictorial warnings was associated with more attention and greater recall [15]. Here, we examine the qualitative findings from in-depth interviews with a subset of these adolescents to understand their reactions to and experiences with pictorial warnings, advancing the science of how pictorial warning labels work.

## Methods

### Participants and procedures

**Fig. 1:**
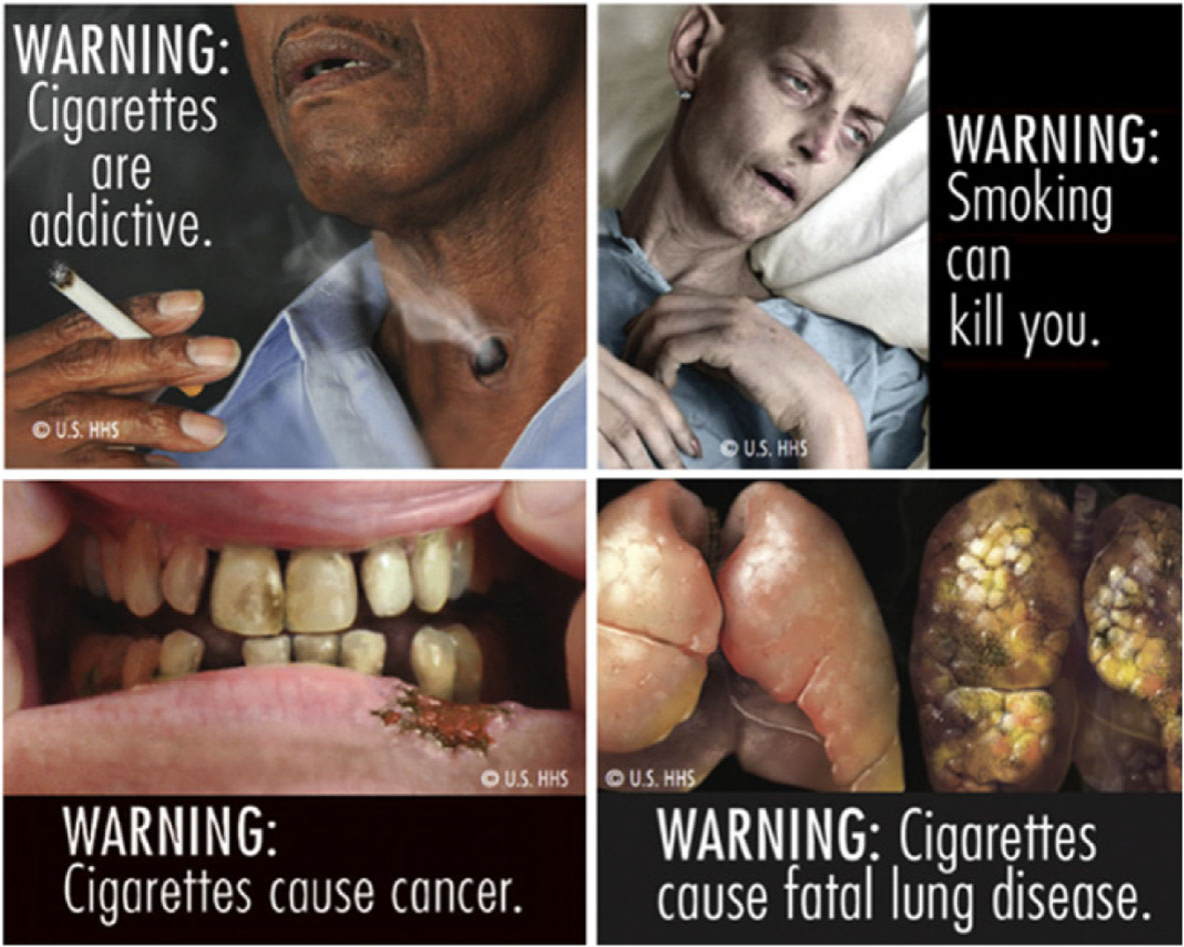
Pictorial warnings used in the randomized clinical trial

From December 2014 to September 2015, we recruited 24 adolescents with a parent, guardian, or other household member who participated in our RCT of pictorial cigarette pack warnings, which enrolled a convenience sample of adult smokers in California and North Carolina, US [16]. The RCT affixed pictorial-and-text versus text-only warning labels to participants’ cigarette packs for 4 weeks [5, 17]. A participant received the same warning for the duration of the study. The four pictorial warnings, that included text and imagery proposed by the US Food and Drug Administration, showed healthy and unhealthy lungs, a person with a tracheotomy, a sick person in a hospital bed, and a diseased mouth (Fig. 1).

We contacted RCT participants who had adolescents ages 13–17, usually the adolescent’s mother or father, referred to hereafter as the “parent.” We obtained verbal consent from the parent for the adolescent’s participation and then verbal assent from the adolescent. We stopped recruitment of adolescents once we reached saturation (i.e., when we stopped hearing new information).

We conducted qualitative interviews by phone with adolescents who reported seeing the pictorial warnings. Both interviewers (KP, MGH) were graduate students trained in qualitative interviewing techniques. Interviewers followed a guide that began with an ice-breaker question about how participants felt about their parents’ smoking and then addressed four content areas: attention to and thoughts about pictorial warnings, emotional reactions to the warnings, conversations sparked by the warnings, and perceptions of warning effectiveness. The interview started with the following instructions: “For the time that we’re on the phone, I’d like to ask that you give me your full attention and not do other things that would distract you, like be on a computer or watch TV. Please also pick a place where you can talk privately.” Additional file 1: Appendix A contains the interview guide used with study participants. The average duration of the interviews was 26 min (range: 15–36 min). We digitally recorded the interviews and an independent company transcribed them. Participants received a $40 incentive for completing the interview. The University of North Carolina’s institutional review board approved the study.

### Data analysis

We conducted a thematic content analysis of the data, creating emergent codes under our pre-specified research questions [18]. We applied Green and Thorogood’s five criteria for qualitative analysis [18]. First, the research team created a codebook built from the questions in the interview guide and then KB and MJB each read and coded the same three transcripts using NVivo Pro v. 11 (QSR International) to create codes relevant to each content area. Next, KB and MJB discussed the results and revised the codebook. KB used the revised codebook to code three new transcripts, and then the coders met to discuss and finalize the codebook. KB then used the final codebook to code all remaining transcripts. Finally, we employed quote matrices to summarize findings and highlight examples of key themes. We organized the findings into the categories of the Tobacco Warnings Model (i.e., attention to warnings, negative emotional reactions, social interactions, and discouragement from smoking), which describes the psychological processes involved in how cigarette pack warnings work [19].

## Results

Most adolescents (*n* = 16) were non-smokers (had never tried cigarettes), five were ever-smokers (had tried cigarettes but had not smoked within the past 30 days), and three were current smokers (smoked within the past 30 days, Table 1). Most participants were Black (*n* = 16) and living in low-income households (*n* = 15). Most adolescents reported seeing their parents’ labeled packs when their parents pulled out cigarettes to smoke or when the packs were left around the house. Two adolescents only viewed the warnings because their parent told them about being in the trial.

**Table 1:** Participant characteristics (*n* = 24)

	Number
Age, mean years (SD)	15 (1.5)
13	5
14	4
15	5
16	4
17	6
Gender	
Male	14
Female	9
Respondent stated “gender-neutral”	1
Ethnicity	
Not Hispanic	22
Hispanic	2
Race	
Asian	2
Black	16
White	4
Other/multiracial	2
Low income household (≤ 150% of Federal Poverty Level)	
No	9
Yes	15
Smoking status	
Non-smoker	16
Ever-smoker	5
Current smoker	3
Study site	
California	11
North Carolina	13
Time between parent’s completion of RCT and adolescent’s interview, mean days (SD)	20 (16)

Nearly all adolescents expressed concern and anxiety about their parents’ smoking. They worried that their parents could become sick or die from smoking-related diseases. Many described watching grandparents or other relatives who smoked die from cancer or lung disease and expressed fear that their parents could experience the same consequences. Teens had extensively thought about and formed opinions about their parents’ smoking. Some felt their parents should stop smoking in order to stay alive and raise their children: “I just wish my dad and my mom’d stop smoking. I need them to be here.” (female non-smoker, age 14). While still communicating concern for their parents’ health, a few of the teens said that they respected their parent’s choice to smoke or explained how their parent needed to smoke to manage their stress. For example, one teen described how smoking served as a coping mechanism for his dad, a veteran: “I mean, he’s been in the war and stuff. . . he’s smokin’ cigarettes for a better reason other than ‘I want to smoke.’” (male ever-smoker, 17).

### Teens paid attention to and thought about pictorial warnings

When we asked about the pictorial warnings, teens described how the warnings caught their attention and were difficult to ignore: “It’s right there. It’s right in front of the box. You can’t miss it.” (male ever-smoker, 16). Teens believed smoking was dangerous before seeing the warnings, but the warnings reinforced their existing beliefs and increased the reality of the health consequences: “It made me think, like, this is really possible. This isn’t fiction.” (male non-smoker, 17). One teen described how the warnings strengthened her negative beliefs about smoking:

You don’t really get to visualize it when someone just says, ‘you can get risks by smoking’ but when you actually see something like [the pictorial warning], you actually get a new perspective . . . it makes you worried and afraid and a little bit scared and nervous that maybe that could happen to you and you just think about how you don’t want that to happen to you and your parents and your family and friends. (female non-smoker, 13).

Like many of the teens, this participant indicated that thinking about the warning label and its depicted health consequences happening to her family and friends stirred up negative emotions.

### Pictorial warnings elicited negative emotional reactions

Teens described negative emotions including fear, disgust, shame, anxiety, and sadness after seeing the pictorial warnings. Most of the adolescents expressed feeling anxious or sad, concerned that the specific health consequence depicted in the warning could happen to them or to their parents. Some teens said that although they had previously seen and heard warnings about smoking, the pictorial images on the cigarette packs were more realistic than other warnings, enabling them to visualize the consequences of smoking and causing a stronger emotional reaction. For example, teens who saw the warning with unhealthy lungs expressed anxiety that their parents’ lungs might look like the lungs on the warning:

. . . that’s probably what my mama’s lungs look like. (male non-smoker, 17).

. . . it made me sad to think what [my dad’s] lungs already look like or what possible disease or cancers he has from smoking. (male non-smoker, 16).

[My mom] could already be developing heart disease or lung cancer or something like that, and you know, that’s something that any kid would be scared of, to see their mother be diagnosed with some, like, crazy disease like that. (male ever-smoker, 16).

Similarly, an adolescent exposed to the sick person warning explained that she had “just like an anxious feeling. ‘Cause you don’t want your parent to get cancer. Especially, like, if it’s something that they can control.” (female non-smoker, 15). Nearly every participant expressed concerns and fears evoked by the warning about their own health or their parents’ health. Teens also described feelings of shame, disappointment, and embarrassment about their parents’ smoking after seeing the pictorial warnings.

Many believed their parents had control over the decision to smoke and were frustrated that their parents continued smoking despite seeing the consequences so clearly depicted on their cigarette packs. For instance, one adolescent said that when her mother took out her cigarettes to smoke, people asked about the image, requiring the mother to explain the picture. The teen expressed frustration and embarrassment that her mother could explain why smoking was dangerous yet continued to smoke. One of the current smokers also reported feeling ashamed about their own personal smoking habits, and said this shame could be productive for quitting:

Whenever I see warning labels, it kind of makes me feel a little ashamed to smoke so many cigarettes. ‘Cause, well, when you smoke a lot of cigarettes for a good amount of time you can feel your lungs and your throat begin to. . . you can feel the, like, smoker’s voice and the smoker’s cough. . . But [being ashamed] also does help because I am trying to quit and it is more motivation. (current smoker who identified as “gender-neutral,” 16).

The negative emotions that resulted from thinking about the warnings seemed to motivate teens to both consider their own behavior and to talk with others about smoking.

### Pictorial warnings sparked conversations

Most of the teens reported at least one instance in which they initiated a conversation with their parents because of the pictorial warning. In these conversations, teens expressed concerns that the health effects on the warnings could happen to the parents. The conversations frequently revolved around the teen’s desire for the parent to stop smoking. One teen described initiating a conversation after he saw his mother’s cigarette pack with a pictorial warning on the kitchen table. He said the image was a sad reminder of his grandfather, who was also a smoker, dying from lung cancer:

I said, ‘Mom I really would like it if you would put that away ‘cause I don’t like seeing it because it bring back bad memories of my grandad passing and I really would like it if you stopped—I mean, if you would stop smoking.’ (male non-smoker, 17).

Teens told their parents that what was on the warning could happen to the parents. In some cases, these conversations provided opportunities for children to discuss their parents’ progress toward quitting. For example, a 17-year-old ever-smoker male said, “I said that’s what your lungs is probably looking like. . . she said she would finally quit. So I’m. . . I think she’s getting the message.” Similarly, a 16-year-old non-smoker male described how he encouraged his mother to cut back on smoking after being exposed to the warnings, saying, “Well she usually tells me how many she smoked… I’m like, ‘Yeah. Good job.’” One girl whose mother had quit smoking by the end of the trial felt the conversation she had with her mother was very influential:

I showed her the picture and I said, ‘That could be, like, you one day, and there could be some serious, maybe even devastating disease.’ . . . She said, “I think that [quitting] it’s a really good idea, because this could be me, and I don’t want this to happen to me at all.” (female non-smoker, 13)

In one case, the warning sparked a conversation focused on why the teen should avoid smoking in the future:

(Interviewer) So what did your grandma think about the warning label?

She told me she hoped I never smoke cigarettes.

(Interviewer) Yeah. Did she say anything about the warning label?

She said my lungs would look like that if I smoke. If I don’t smoke, my lungs would be healthy. (female current smoker, 14).

Several teens reported talking to their friends about smoking after seeing the warnings. For instance, one teen talked about showing the warning to his friend to encourage him to quit smoking:

My friend just kind of recently started smoking so I wanted him to see the pack. And when I did show him the pack of cigarettes, it made him kind of think about it and he kind of like put the pack down for a little bit once he seen it . . . seeing that skinny man, I guess, just like me, made him sad and made him like, ‘well I don’t want to become or look like that.’ (male non-smoker, 17).

In sum, the pictorial warnings sparked numerous conversations between adolescents and their family members as well as their friends. These conversations often focused on the powerful images and the need to avoid or quit smoking.

### Teens largely thought pictorial warnings were effective at discouraging smoking

Teens felt that pictorial warnings would help discourage them and others from smoking. Non-smoking teens said the warnings reinforced their decision not to smoke:

It has really made me think that I definitely don’t want to smoke at all seeing that cigarette pack. I don’t want to be around the smoke, I don’t want to smoke, I don’t want nobody I love to smoke. (male non-smoker, 17).

I guess definitely it holds me back from smoking. It doesn't make me want to smoke at all, especially because I do athletics, too, and once I see the lungs, which I actually saw, it was just like wow, I really don't want to smoke at all. It's nasty to me, to be honest. (male non-smoker, 16).

Two of the current smokers described how the warnings made them reconsider their own smoking. One talked about how the warnings were an “eye-opener,” (male current smoker, 15) and the other discussed how seeing the warnings added to previous messages: “I’ve seen warning labels like that before, but I guess the more I see them, the more I’m consciously aware that that’s what I’m doing to myself.” (current smoker who identified as “gender-neutral,” 16). When asked how the warning changed how she thought about smoking, another current smoker indicated that it made her want to quit smoking: “Just the way it looked. . . I don’t ever want to smoke.” (female current smoker, 14). The visual imagery resonated with the teens and appeared to heighten their awareness of the seriousness of the risks involved.

Most adolescents felt that the warnings would convince smokers to quit and prevent people, especially teens, from starting to smoke. For example, a 17-year-old male who had recently quit after 5 years of smoking said that if his very first pack of cigarettes had displayed a similar picture, he probably would not have started smoking. He said the warnings allow someone to make a more conscious decision about smoking:

. . . it kind of gives people a heads up so they’re not walking into war with a blindfold on. It’s kind of like helping people out in a way. Just it’s a fair warning: ‘Hey look, go ahead and smoke this but this could happen if you keep smoking it.’ (male ever-smoker, 17).

Some adolescents said that the pictorial warnings would have more impact than the current Surgeon General warnings:

Because you know how every pack has a warning label, that’s a warning label, but it doesn’t really give your mind the image. The actual image that shows you what can happen. It may give you that extra push, like, I don’t want to smoke a cigarette. (male non-smoker, 17).

A few teens, however, expressed doubts about the warnings’ effectiveness. For instance, one 15-year-old current smoker said that the warning would not have prevented him from starting to smoke at age 12 because he “had it pretty hard growing up” so he “didn’t really care.” Another current smoker found the warning personally effective, but did not think the warning would affect her parents: “They’d just look at the pack, and [think] like ‘that ain’t nothing to me. My lungs is already black.’” One teen mentioned potential limitations to the effectiveness of the warnings for smokers, given the addictiveness of cigarettes:

Well, I think it could potentially stop some people from smoking, but I think that if someone has already started to smoke, then the warning probably won’t make them stop smoking. I mean that it’s a very addictive thing. (male non-smoker, 13).

Similarly, a few teens said that most smokers already know that smoking has negative consequences, so viewing the warning may not produce a change. The teens largely supported the idea of the government requiring pictorial warnings, although one 13-year-old non-smoker male thought that cigarette companies should be able to decide for themselves how to label packages: “That’s cigarette companies’ own business. . . they’re allowed to do what they want. But I do think it can be helpful. It can stop people from smoking. I don’t know how often, but I do think it can.” Despite expressing some doubts, teens generally believed that pictorial warnings would be worthwhile even if they did not deter smoking in every case.

## Discussion

In interviews with US adolescent children of smokers, we found that teens worried about their parents’ smoking, and pictorial cigarette pack warnings heightened this worry. Teens paid attention to the pictorial warnings and experienced strong negative emotional reactions, which typically resulted from thinking about the health consequence depicted on the warning happening to their parent or to them. The pictorial warnings also inspired numerous conversations with family and friends about quitting smoking. Most of the teens in the study believed that the pictorial warnings would be effective. Several said that the warnings reinforced their existing negative impression of smoking and some of the smokers said they made them consider quitting.

Teens reported feeling anxiety, sadness, disgust, and shame in response to seeing the warnings. Prior research shows that negative emotions are an important part of how pictorial warnings exert their effects [20–22], acting as a mediator between the warnings and outcomes such as intentions and behavior [19, 23–25]. The current study extends these findings by describing the natural language adolescents use to describe the emotions they experience in response to pictorial warnings. It also provides insight about why adolescents experience these negative emotions when they view these warnings; namely, adolescents visualize the health effects shown in the image as happening to themselves, their parents, or other people they know who smoke.

We found that pictorial warnings cued conversations among adolescents [15], building on previous research that pictorial warnings spark conversations among adults [5, 26–28] (Morgan JC, Golden SD, Noar SM, Ribisl KM, Southwell BG, Jeong M, Hall MG, Brewer NT: Conversations about pictorial cigarette pack warnings: Theoretical mechanisms of influence impact quit attempts, submitted). Pictorial warnings offered an important opportunity for adolescents to broach the topic of smoking cessation with their parents (and sometimes with their friends). In these conversations, teens expressed their fears and frustrations with their parents and encouraged them to quit smoking [29]. Researchers have described teen-initiated conversations about smoking as “parenting the parent.” In a qualitative study of Canadian youth, researchers found that expressing concerns that the parent might die was a strategy used by teens to encourage their parents to quit smoking. In contrast to that study, which found that most youth who initiated these conversations did not feel their efforts were worthwhile, in our study many teens thought that expressing their concerns would help parents quit. It may be that the pictorial warnings legitimate and make the concerns more concrete, potentially enhancing the effectiveness of teens’ conversations. Considering the addictiveness of cigarettes [30], however, future research should explore the impact of conversations about pictorial warnings initiated by adolescents on subsequent quitting among parents and friends.

To our knowledge, ours is the first qualitative study about how US adolescent children of smokers react to pictorial warnings. Additionally, our study examined how teens reacted to pictorial warnings in their own natural environment rather than in a laboratory setting. Furthermore, our sample included a diverse group of teens with respect to race and poverty, although the generalizability of our findings remains to be established. Another limitation is that our sample contained only three current smokers; future research should examine reactions to pictorial warnings in a larger sample of adolescent smokers. We also conducted the interviews by phone, which may have reduced the potential for rapport between interviewers and participants. Finally, because smoking is socially stigmatized, participant responses may not have been fully candid.

## Conclusions

Adolescents who viewed pictorial warnings on cigarette packs felt negative emotions and visualized the health consequences of smoking. The warnings provided an important opportunity for adolescents to talk to their parents about quitting smoking. This finding suggests that pictorial warnings may have a broad reach, affecting not only smokers but also their children and potentially others in their social networks. The present study adds to the large body of evidence supporting pictorial warnings as a meaningful policy tool to reduce tobacco use.

## Additional file

Additional file 1: Appendix A. Interview guide. This file contains the interview guide used with study participants. (DOCX 22 kb)

## Abbreviations

RCT: Randomized clinical trial
US: United States

## References

[1] Mays D, Gilman SE, Rende R, Luta G, Tercyak KP, Niaura RS (2014). Parental smoking exposure and adolescent smoking trajectories. Pediatrics.

[2] Kandel DB, Griesler PC, Hu M-C (2015). Intergenerational patterns of smoking and nicotine dependence among US adolescents. Am J Public Health.

[3] Chassin L, Presson C, Rose J, Sherman SJ, Prost J (2002). Parental smoking cessation and adolescent smoking. J Pediatr Psychol.

[4] Noar SM, Hall MG, Francis DB, Ribisl KM, Pepper JK, Brewer NT (2016). Pictorial cigarette pack warnings: a meta-analysis of experimental studies. Tob Control.

[5] Brewer NT, Hall MG, Noar SM (2016). Effect of pictorial cigarette pack warnings on changes in smoking behavior: a randomized clinical trial. JAMA Intern Med.

[6] Noar SM, Hall MG, Brewer NT (2015). Pictorial cigarette pack warnings have important effects. Am J Public Health.

[7] Hammond D (2011). Health warning messages on tobacco products: a review. Tob Control.

[8] Moodie C, Mackintosh AM, Hastings G (2015). Adolescents’ response to pictorial warnings on the reverse panel of cigarette packs: a repeat cross-sectional study. Tob Control.

[9] Vardavas CI (2009). Adolescents perceived effectiveness of the proposed European graphic tobacco warning labels. Eur J Pub Health.

[10] White V, Webster B, Wakefield M (2008). Do graphic health warning labels have an impact on adolescents smoking-related beliefs and behaviours?. Addiction.

[11] McCool J, Webb L, Cameron LD, Hoek J (2012). Graphic warning labels on plain cigarette packs: will they make a difference to adolescents?. Soc Sci Med.

[12] Andrews JC, Netemeyer RG, Burton S, Kees J (2016). Effects of plain package branding and graphic health warnings on adolescent smokers in the USA, Spain and France. Tob Control.

[13] Evans AT, Peters E, Shoben AB, Meilleur LR, Klein EG, Tompkins MK, Romer D, Tusler M (2017). Cigarette graphic warning labels are not created equal: they can increase or decrease smokers’ quit intentions relative to text-only warnings. Nicotine Tob Res.

[14] Netemeyer RG, Netemeyer RG, Burton S, Andrews JC, Kees J (2016). Graphic health warnings on cigarette packages: the role of emotions in affecting adolescent smoking consideration and secondhand smoke beliefs. J Public Policy Mark.

[15] Peebles K, Hall MG, Pepper JK, Byron MJ, Noar SM, Brewer NT (2016). Adolescents’ responses to pictorial warnings on their parents’ cigarette packs. J Adolesc Health.

[16] Brodar K, Hall M, Butler E, Parada H, Stein-Seroussi A, Hanley S, Brewer N (2016). Recruiting diverse smokers: enrollment yields and cost. Int J Environ Res Public Health.

[17] Brewer NT, Hall MG, Lee JGL, Peebles K, Noar SM, Ribisl KM (2015). Testing warning messages on smokers’ cigarette packages: a standardised protocol. Tob Control.

[18] Green J, Thorogood N (2009). Qualitative methods for health research.

[19] Brewer NT, Parada H, Hall MG, Boynton MH, Noar SM, Ribisl KM. Understanding why pictorial cigarette pack warnings increase quit attempts. Ann Behav Med. 2018. Advance online publication. 10.1093/abm/kay032. 10.1093/abm/kay032 PMC6265120 29850764

[20] Cameron LD, Pepper JK, Brewer NT (2013). Responses of young adults to graphic warning labels for cigarette packages. Tob Control.

[21] Wang A-L, Lowen SB, Romer D, Giorno M, Langleben DD (2015). Emotional reaction facilitates the brain and behavioural impact of graphic cigarette warning labels in smokers. Tob Control.

[22] Kees J, Burton S, Andrews JC, Kozup J (2010). Understanding how graphic pictorial warnings work on cigarette packaging. J Public Policy Mark.

[23] Hall MG, Sheeran P, Noar SM, Boynton MH, Ribisl KM, Parada H, Johnson TO, Brewer NT. Negative affect, message reactance, and perceived risk: how do pictorial cigarette pack warnings change quit intentions? Tob Control. 2017. Advance online publication. 10.1136/tobaccocontrol-2017-053972. 10.1136/tobaccocontrol-2017-053972 PMC6004228 29248897

[24] Emery LF, Romer D, Sheerin KM, Jamieson KH, Peters E (2014). Affective and cognitive mediators of the impact of cigarette warning labels. Nicotine Tob Res.

[25] Evans AT, Peters E, Strasser AA, Emery LF, Sheerin KM, Romer D (2015). Graphic warning labels elicit affective and thoughtful responses from smokers: results of a randomized clinical trial. PLoS One.

[26] Hall M, Peebles K, Bach L, Noar S, Ribisl K, Brewer N (2015). Social interactions sparked by pictorial warnings on cigarette packs. Int J Environ Res Public Health.

[27] Thrasher JF, Abad-Vivero EN, Huang L, O'Connor RJ, Hammond D, Bansal-Travers M, Yong H-H, Borland R, Markovsky B, Hardin J (2016). Interpersonal communication about pictorial health warnings on cigarette packages: policy-related influences and relationships with smoking cessation attempts. Soc Sci Med.

[28] Morgan Jennifer C, Southwell Brian G, Noar Seth M, Ribisl Kurt M, Golden Shelley D, Brewer Noel T (2017). Frequency and Content of Conversations About Pictorial Warnings on Cigarette Packs. Nicotine & Tobacco Research.

[29] Woodgate RL, Kreklewetz CM (2012). Youth’s narratives about family members smoking: parenting the parent- it’s not fair. BMC Public Health.

[30] Hyland A, Li Q, Bauer JE, Giovino GA, Steger C, Cummings M (2004). Predictors of cessation in a cohort of current and former smokers followed over 13 years. Nicotine Tob Res.

