# Supplementary material for: “That’s probably what my mama’s lungs look like”: how adolescent children react to pictorial warnings on their parents’ cigarette packs
Source: BMC Public Health. 2018 Sep 15;18:1125. doi: 10.1186/s12889-018-6011-7 (PMC6139175; doi:10.1186/s12889-018-6011-7)
Supplement: Supplementary file 1 — Appendix A. Interview guide. This file contains the interview guide used with study participants. (DOCX 22 kb) [file 12889_2018_6011_MOESM1_ESM.docx]

**Appendix A.** Interview guide

| **1. Here is my first question: Teens we’ve talked to have all kinds of opinions about smoking. What are your thoughts about smoking in general?**  *Probe:* That’s very interesting. Can you tell me more about that?  *Probe:* How do you feel when you see someone smoking?  *Probe:* How do you feel about [RCT participant’s] smoking? |
| --- |
| **2. Earlier we were talking about warning labels on [RCT participant’s] cigarette packs. Think about a time you saw the warning label.**  *Probe:* Where were you?  *Probe:* What were you doing?  *Probe:* Did you pick it up?  *Probe:* How long did you look at it for?  *Probe:* When do you usually see the warning label? |
| **3. Tell me what you thought about the warning label.**  *Probe:* How did the warning label make you feel?  *Probe*: Earlier, you mentioned that the label made you feel [emotion from survey]. Tell me more about that. |
| **4. How has seeing the warning label changed how you think about smoking, if at all?**  *Probe*: It’s fine if you haven’t thought about this before. Looking back now, what effect do you think it’s had? |
| **5. What did [RCT participant] think about the warning label?**  *Probe*: Did you talk about it? Who started the conversation?  *Probe*: What did you talk about?  *Probe:* How do you think the warning label affected [RCT participant’s] smoking?  *Probe*: Who else did you talk to about the label? What did you talk about? |
| **6. So you told me earlier that the warning label on [RCT participant’s] cigarette packs had a picture of [description of warning label]. What other anti-smoking warnings have you seen?**  *Probe: [If participant can’t think of any warnings]* For example, have you seen any anti-smoking ads on TV or heard any on the radio?  *Probe:* Tell me about those warnings. How did they make you feel?  *Probe*: What did you think about the warning label on [RCT participant’s] cigarette packs compared to those other warnings?  *Probe*: How was the warning on your mom’s cigarette packs different? How was it the same? |
| **7. Earlier, you told me that you thought the US [should/should not] require picture warnings on all cigarette packs. Tell me a bit about why you think that.** |
| **8. Let’s imagine you’re in charge of designing cigarette pack warning labels that will make people not want to smoke. If you could do anything you wanted, what would the warning labels look like?**  *Probe:* Would your label have just a picture, just words, or both? ... Ok, tell me why you would make it that way.  *Probe:* That sounds like a great warning label. Tell me about how you think teens would react to it.  *Probe:* How would you want smokers to feel when they see the label?  *Probe:* What types of pictures do you think would make people not want to smoke? What would the warnings say?  *Probe*: Thinking about the warning label on [RCT participant]’s cigarette packs, how could we make that label better? |
| **9. Thank you. Those are all the questions I have. Is there anything else you’d like to say or that you think we should know?** |
